# Using approach latency and anticipatory behaviour to assess whether voluntary playpen access is rewarding to laboratory mice

**DOI:** 10.1038/s41598-021-98356-3

**Published:** 2021-09-21

**Authors:** Anna S. Ratuski, I. Joanna Makowska, Kaitlyn R. Dvorack, Daniel M. Weary

**Affiliations:** Animal Welfare Program, Faculty of Land and Food Systems, University of British Columbia, 2357 Main Mall, Vancouver, BC V6T 1Z6 Canada

**Keywords:** Zoology, Animal behaviour, Behavioural methods, Model vertebrates

## Abstract

Laboratory mice are typically housed in “shoebox" cages that limit the expression of natural behaviours. Temporary access to more complex environments (playpens) may improve their welfare. We aimed to assess if access to playpens is rewarding for conventionally-housed mice and to document mouse behaviour during playpen access. Female C57BL/6J, BALB/cJ, and DBA/2J mice were provided temporary access to a large enriched playpen three times per week; control mice remained in their home cages. We measured latency to enter playpens and anticipatory behaviour to determine if access was rewarding, and recorded mouse behaviour during playpen sessions. Over time, playpen mice entered the playpen more quickly; latency declined from 168 ± 22 to 13 ± 2 s over the 14-d trial. As expected, playpen mice showed an increase in anticipatory behaviour before playpen access (mean ± SE = 19.7 ± 2.6 behavioural transitions), while control mice showed no change in anticipatory behaviour relative to baseline values (2.4 ± 1.6 transitions). Mice in the playpen performed more ambulatory behaviours than control mice who remained in home cages (21.5 ± 0.7 vs 6.9 ± 1.1 observations of 25 total observations). We conclude that conventionally-housed mice find voluntary playpen access rewarding, and suggest this as a useful option for providing laboratory mice with access to more complex environments.

## Introduction

Laboratory mice (*Mus musculus*) are usually housed in ‘shoebox’ cages that contain bedding, food, water and occasionally nesting material or shelter. The lack of space and complexity in conventional housing restricts natural behaviours such as running, climbing, and burrowing^1^. Many natural behaviours remain important for laboratory mice even though they have been purpose-bred and housed in restricted conditions for generations. For example, laboratory mice will work for access to nesting material^2^, and when provided with an appropriate substrate will begin to burrow almost immediately^3,4^.

There is ample evidence that conventional housing negatively affects mouse physiology and behaviour, raising concerns about animal welfare and scientific validity (e.g.^5,6^). Without the ability to express natural behaviours, mice show signs of poor welfare, including negative affective states^7,8^. Laboratory mice housed in conventional cages commonly develop behavioural stereotypies (i.e. repetitive, unvarying behavioural patterns), which can indicate poor welfare^9,10^, and show high levels of inactive-but-awake behaviour, potentially indicative of a depressive state^11^. There are also negative physiological and neurological impacts of conventional cages, such as increased pain responses^12^, increased disease susceptibility related to obesity and lack of exercise^13^, and reduced neuroplasticity (e.g.^14^).

Environmental enrichment can allow for the expression of a wider variety of natural behaviours^2^. The welfare benefits of enrichment are enhanced through the use of more complex environments; mice housed in larger cages with a variety of enrichment (i.e. climbing structures, tunnels, shelters, nesting material, elevated platforms and bridges) engage with enrichment materials more frequently than mice housed in conventional cages with basic enrichment objects (i.e. nesting material, a tunnel, and a shelter^15^). Furthermore, mice housed with more extensive enrichment display virtually no stereotypic behaviours^9,15^.

Despite the scientific advances, little progress has been made in changing housing conditions for laboratory mice. Implementation of extensive enrichment in home cages may be impractical for some research facilities due to space requirements, cost, sanitation, and animal visibility^15^. Temporary access to a more complex environment (e.g. with playpens) may offer some benefits and could be more practical for some facilities. Mouse playpens can be crafted using sterilized rat cages and a variety of substrates and objects, and the same playpen can be used for several groups of mice. Rats show anticipation before transfer into enriched cages for 30 min; this anticipatory response is similar to that shown prior to sexual contact, suggesting that access to temporary enrichment is rewarding^16^. Mice show anticipatory behaviour before accessing food rewards^17^, and show motivation to access an enriched cage attached to their home cage via a weighted door^18^. Latency to approach can also be used as a measure of reward^17,19^.

To date, no studies have examined if access to playpens is rewarding for mice. There are several reasons why playpens may not be rewarding: mice may be initially fearful of enrichments due to neophobia^20^; mice experiencing anhedonia (i.e. a depressive-like state) are less willing to explore novel environments^21^; highly valued enrichment objects may lead to increased competition and aggression^22^; and environmental enrichment may actually increase anxiety for some strains of laboratory mice^23^. Therefore, the first aim of the current study was to assess if access to larger and more complex environments (playpens) is rewarding for mice.

The secondary aim of this study was to document mouse behaviour in playpens to determine how these are used by the animals. Within the literature on mouse enrichment there is much variation in the components provided, and animal engagement with environmental components is sometimes not assessed. Additionally, the welfare problems of inactivity or under stimulation have been previously highlighted as an area where research is lacking^2^. One of our goals in setting up the playpen was to create an engaging environment that increased behavioural opportunities, so we aimed to assess how mice interacted with the provided items and how the playpens affected overall activity levels.

We predicted that, over time, mice with playpen access would show increased anticipatory behaviour before playpen access and would enter playpens more quickly, indicating that playpen access was rewarding. We also predicted that mice would engage with the provided items in the playpen, resulting in a wider range of ambulatory behaviours while in playpens. During the study, in which three different strains of mice had access to the playpen at the same time, it became apparent that the majority of agonistic interactions were instigated by C57BL/6J mice. Therefore, we also documented the frequency of agonistic interactions with and without the C57BL/6J mice in the playpen. We predicted that there would be fewer agonistic social interactions per mouse without the C57BL/6J.

## Methods

This experiment was approved by the University of British Columbia Animal Care Committee (protocol A18-0104). It was performed in accordance with the Canadian Council on Animal Care and ARRIVE guidelines.

### Subjects and materials

Three common strains of female mice (C57BL/6J, DBA/2J, and BALB/cJ, hereafter referred to as C57, DBA, and BALB) were obtained from Jackson Laboratories (Sacramento, California, USA). Mice (n = 42) arrived at the facility at 4 weeks of age and were divided into 14 cages, each with three mice (one from each strain). Housing mice in this way allows for non-invasive identification of individual mice, increases statistical power, and provides a means to examine differences between common strains^24^.

Mice were housed in conventional ventilated cages measuring 32.5 cm long, 17 cm wide, and 14 cm high (Ehret, Germany) that contained aspen chip bedding (Jamieson’s Pet Food Distributors LTD, BC, Canada), nesting material (cotton nestlet, Ancare, NY, USA; Enviropak nesting material, Datesand, UK), a polycarbonate hut (Bio-Serv, NJ, USA), and ad libitum access to irradiated food (Lab Diet Rodent Chow 2918) and reverse osmosis tap water. Mice were kept on a 12-h light and dark cycle (lights on at 7:00 a.m.) with a room temperature of (mean ± SD) 20.7 ± 0.1 °C and humidity of 44.2 ± 4.3%. Cage changes were performed every two weeks by one of the experimenters (mice were transferred using their own inverted hut).

**Figure 1:**
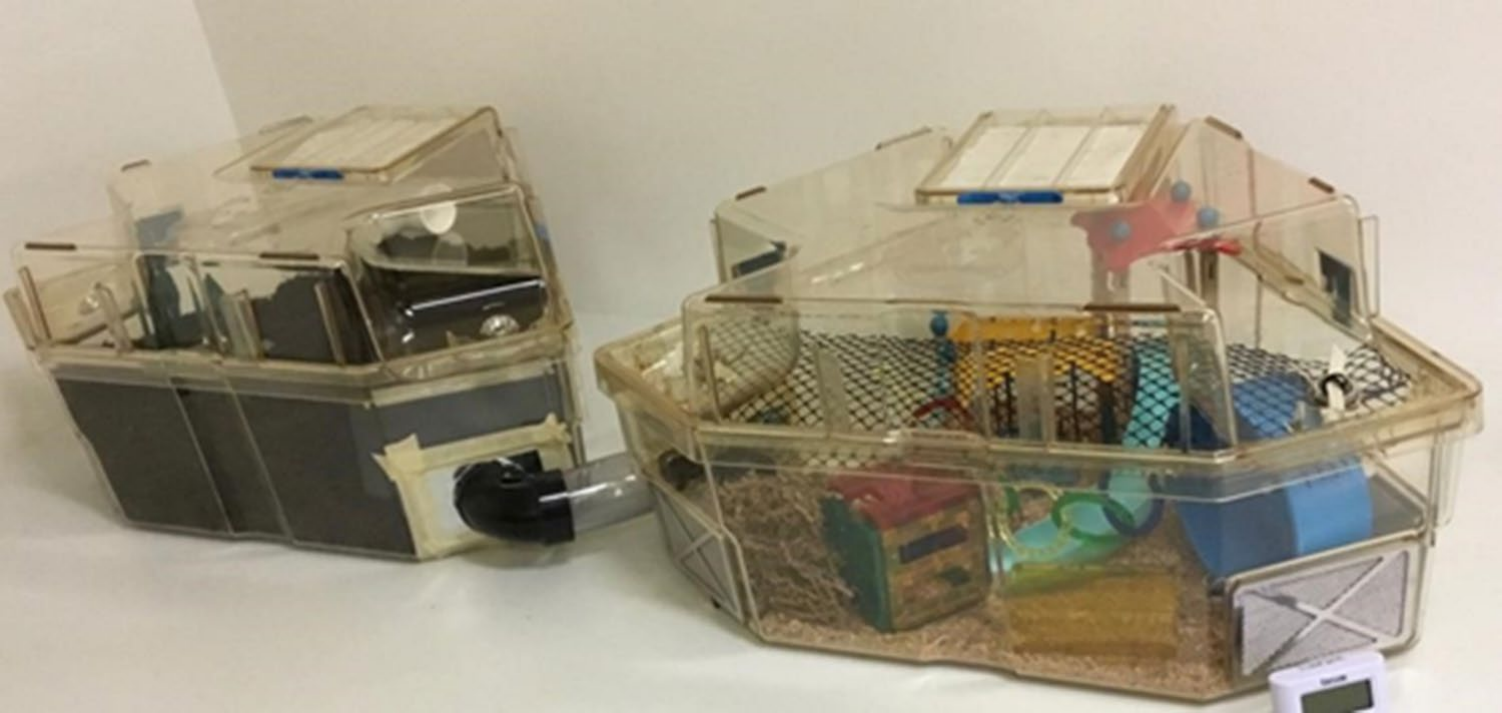
Playpen design. Two rat cages were connected via a clear tunnel. The cage on the left side contains burrowing substrate, a triangular shelter made of black corrugated plastic positioned within the substrate, and a plastic upper mezzanine with an opaque tunnel. The cage on the right contains structural enrichment items: a wheel (14 cm diameter; Kaytee Comfort Wheel, Petsmart, Canada), plastic tunnel (9.8 cm long, Bio-Serv), climbing and shelter structures (Playmobil Park Playground; cube structure made from magnetic PicassoTiles; USA), wood chip bedding, paper nesting material (Enviro-Dri), and plastic netting and hoops suspended from the lid for climbing. Photo provided by ASR.

The playpen set-up (Fig. 1) was designed to provide mice with space and structures that would facilitate a variety of behaviours such as climbing, burrowing, and running. The playpen consisted of two Optirat Plus cages (38.9 cm L × 56.9 cm W × 26.2 cm H, Animal Care Systems, CO, USA) connected together by a PVC tunnel; the tunnel protruded into each cage through a hole that was made by removing the filter. The playpen was not connected to a ventilated cage rack.

One cage of the playpen contained an assortment of structural items, while the other cage contained burrowing substrate approximately 15 cm deep (Fig. 1). Two playpens were used, identical in all ways except the burrowing substrate in one consisted of soil (Garden Club 3-in-1 Mix consisting of humus, sphagnum peat moss, and compost) and the other consisted of coconut fibre (Thrive Natural Compressed Coconut Fiber Reptile Bedding). We included two different substrates for practical purposes: to monitor if they retained their quality over time and whether there was any difference in the tunnels formed by the mice depending on substrate. To control for any possible differences in how the mice perceived the different substrates, they were equally exposed to both by alternating the substrate for each trial. Both substrates were autoclaved before the start of the experiment and periodically watered to keep from becoming too dry to form burrows. The playpens were not cleaned for the entirety of the study; we had planned to clean them if they became visibly soiled, but the mice did not use the playpens as a latrine. After 30 min in the playpens, mice were transferred back to their home cage using a transfer tunnel (All Living Things Tiny Tales transport tubes, Petsmart, Canada) or gentle hut handling if mice did not use the tunnel within 5 min. Autoclaved pumpkin seeds and shredded coconut were provided as a treat in the home cage upon return from the playpen.

### Study design

A pilot study with a separate group of animals was first conducted to determine if mice were willing to use playpens during the light period, which enrichment objects they found engaging, and to refine the methods used for data collection in the main study (see Supplementary Methods for more information). On the basis of these findings, we designed the current study.

Cages were randomly assigned to the playpen treatment (n = 7 cages) or the control treatment (n = 7 cages); thus each treatment consisted of 21 mice (7 mice per strain). Cage placement on the ventilated rack was balanced between treatments to control for differences in light exposure. Playpen mice were given access to playpens for 30 min, three times per week beginning at 42 days of age (± 3 days) until they reached 100 days of age (± 3 days). One of the reoccurring playpen days was scheduled in the late morning and the other two in the early afternoon. Cages were always given playpen access one at a time and in the same order.

### Habituation and training

All mice were habituated to the experimenters for one week before the experiment started. This was done by the experimenters passively placing their hand in the cage and feeding the mice autoclaved pumpkin seeds and unsweetened shredded coconut. At this time, playpen mice were also habituated to the tunnels that were used to transfer mice back and forth between home cages and playpens (see “Data collection” section, and Supplementary Table S1).

To obtain a baseline for anticipatory behaviour, mouse cages were first habituated to placement on the cart for 1 min on two separate days; no auditory cue or playpen access was given at this time—all cages were returned to the cage rack after 1 min. A baseline of anticipatory behaviour was recorded when the mice were 42 ± 3 days old, on the first day of playpen access for mice in the playpen treatment (i.e. to establish a baseline reaction to the cue before mice learned to associate this with the playpen). Training then occurred 3 days per week for 2 weeks. Each day the interval between the cue and the reward was increased by 10 s until the maximum waiting time of 1 min was reached (Supplementary Table S2).

During training and testing trials for anticipatory behaviour, the cage was placed on a cart for 1 min before the filter top, wire lid and water bottle were removed, and an overturned wire lid was placed on top of the cage to allow for increased visibility. Then, the experimenter dragged her finger over the wire lid three times, which served as the auditory cue. The experimenter then stood facing away from the cage during the waiting period to reduce the risk that human gaze would influence mouse behaviour. Once a cue-reward interval of 1 min was reached, training was complete.

### Data collection

#### Anticipatory behaviour

Anticipatory behaviour was assessed using the same methods used during training: each cage was placed on a cart and provided with a conditioned auditory cue that was followed by playpen access for the playpen mice, or the cage was placed back on the cage rack for the control mice. The methods and ethogram used to measure anticipatory behaviour were adapted from Spangenberg and Wichman^17^. Baseline anticipatory behaviour was video recorded for one min on day 1 of playpen access (baseline; before mice had ever accessed the playpen), and again on days 8 to 14 of playpen access (to assess changes from baseline).

An observer blind to cage identity, treatment, and playpen day scored the behaviour of all mice during the 1-min cue-reward interval using an ethogram (Table 1). Interobserver reliability with a second blinded observer was assessed for our outcome measure (total number of behavioural transitions/min) using Pearson correlation with a randomly selected subset of 24 videos (r = 0.96).

**Table 1:** Ethogram used for anticipatory behaviour analysis.

Behaviour	Description
In hut	Head and body are inside hut
On hut	All four paws on top of hut
Locomotion	Moves forward, all paws moving
Sitting	All paws on the ground, not moving forward or backward, may be sniffing
Rear	Both front paws and upper body raised, unsupported or leaning on wall
Rear move	Moves both raised front paws from one position to another
Rear hut	Standing on hut with front paws and upper body raised
In nest	Head and body are covered by nesting material
Social sniff	Nose contacts another mouse
Stretched posture	Upper body stretched forward and raised, posterior body is low to ground
Nesting	Modifying or burrowing in nest material
Out of view	Unable to see mouse and identify behaviour

#### Latency to enter playpen

Once anticipatory behaviour training/testing was complete, the home cage was placed next to the playpen, the lid was removed, the home cage hut was overturned and an extended tunnel (All Living Things Tiny Tales transport tubes) was placed with one end in the home cage and the other in the playpen. Mice could move back and forth between their home cage and the playpen for 5 min. If mice did not enter the playpen within 5 min, they were gently transferred to the playpen using the overturned hut; mice were never tail handled.

The 5-min transfer period was video recorded and this was analyzed by an observer blind to playpen day and cage identity. Latency to enter was scored from the moment the tunnel was placed in the home cage until each individual first entered the playpen. A mouse was considered to have entered the playpen when all four feet exited the tunnel onto the playpen floor. Latency to enter was assessed on days 1, 3, 6, 9, 12, and 14 of playpen access. Interobserver reliability with a second blinded observer was assessed using Pearson correlation for two of the days (42 videos, r = 0.99).

#### Behavioural observations

Live instantaneous scan sampling was performed by the same experimenter once per week for 5 weeks; interobserver reliability was not measured for this outcome and the observer could not be blinded to treatment. The behaviours of playpen and control mice were recorded following the ethogram in Table 2. Observations began approximately 10 min after anticipatory behaviour testing was completed for each cage. Control mice were observed in their home cage on the ventilated rack and playpen mice were observed in the playpens. The behaviour of each mouse was sampled a total of 5 times per observation day and each observation was spaced 2–5 min apart, for a total of 25 observations per mouse. In addition to behaviour, the location of each mouse was noted. For playpen mice the options were left cage (burrowing substrate) or right cage (structural items); for control mice, location options were in the nest, in the hut, or elsewhere.

**Table 2:** Ethogram used for scan sampling and focal sampling observations.

	Description	Activity category
**(a) Scan sampled behaviour**		
Affiliative grooming	Licks fur of another mouse or grooms with front paws, includes both giving and receiving grooming. Recipient mouse is not pinned or held down by groomer	Non-ambulatory
Chewing	Bites on an object other than food or cage bars	Non-ambulatory
Eating or drinking	Mouse drinks from water bottle or gnaws on food in hopper or cage floor	Non-ambulatory
Grooming	Licks, scratches, or manipulates fur	Non-ambulatory
In shelter, out of view	Unable to see mouse and identify behaviour; in control cages this meant the mouse was fully inside the nest or the hut, while in the playpen this meant the mouse was inside the structure under the burrowing substrate	Non-ambulatory
Nesting	Modifying or manipulating any nesting material with paws or mouth	Non-ambulatory
Resting	Sitting or sleeping alone or with other mice, no movement	Non-ambulatory
Sniff	Mouse is seated or immobile with nose elevated and sniffing, or nose contacts another mouse	Non-ambulatory
Stretched/alert posture	Head of mouse is raised, appears alert, body remains low to ground	Non-ambulatory
Agonistic interactions	Encompasses any agonistic behaviours, including chasing, fighting, pinning, anogenital sniffing, and mounting	Ambulatory
Climbing	Body is suspended from the wire lid, plastic netting, or vertical playground structure in the playpen; mouse is held up by two or four paws, all four paws are off the floor	Ambulatory
Digging	Mouse engages front and/or back legs in manipulating burrowing substrate or cage bedding	Ambulatory
Frisky	Sudden bouncy hops, skips or erratic locomotion	Ambulatory
Harassed	Mouse is recipient of agonistic behaviour such as being attacked, mounted, pinned, or chased by another mouse	Ambulatory
Rear	Mouse is on hind paws with both front paws and upper body raised	Ambulatory
Running	Moves forward locomotion at a fast pace with all paws moving	Ambulatory
Stereotypic behaviour	Includes all identifiable stereotypies such as backflipping, bar biting, route tracing, or repetitive circling or twirling on the cage lid; descriptions based on^28^	Ambulatory
Swing	Mouse has at least two paws on swing and the swing is in motion	Ambulatory
Walking	Moves forward walking, locomotion at a slow or moderate pace	Ambulatory
Wheel use	Moves all paws while on the wheel	Ambulatory
**(b) Agonistic behaviours scored on days 30–33**		
Pinning/boxing	Mouse pins down another mouse or mice are pushing each other with forearms	
Chasing	Mouse pursues another mouse	
Rough grooming	Mouse pins down another mouse and vigorously grooms hair	
Mounting	Mouse attempts to mount and perform pelvic thrusts on another mouse	
Displacement	Mouse pushes or supplants another mouse from a resource	
Anogenital investigation	Mouse persistently pushes or sniffs another mouse’s anogenital region	
Fighting	Mice are locked together rolling around quickly, kicking, biting, or wrestling; mice involved in fights will both be labeled as aggressor	
Receiving aggression	Mouse is the recipient of any forms of aggression listed above	

(a) The ethogram for scan sampling observations is listed first and was adapted from Makowska et al.^26^ and Draper^27^. (b) For focal sampling of agonistic interactions of the playpen mice on days 30–33, only agonistic behaviours listed in the lower half of the table were scored (adapted from Nip et al.^11^).

In early trials we observed that antagonistic interactions in the playpens were largely initiated by the C57 mice, so we hypothesized that fewer antagonistic interactions would be observed if C57 mice were absent. To test this hypothesis, mice were observed in the playpen during four sessions—on playpen days 30–33—with the presence or absence of the C57 mouse alternating across sessions (i.e. two sessions with and two without; the C57 mouse was still given access to the playpen (by herself) on days she was not tested with her cage mates). During these sessions, each cage was observed using focal sampling^25^ by the same observer in alternating 5-min periods, for a total of 15 min of observation per day for four days. During these observations only agonistic behaviours were scored and all occurrences were recorded. Observations began after mice had been in the playpens for 5 min. Agonistic interactions were scored using an ethogram from Nip et al.^11^ (Table 2). On these days, all mice were handled using the overturned hut from the home cage rather than the tunnel to ensure mice entered the playpen at the same time.

### Statistical analysis

Statistical analyses were performed using SAS software (Version 9.4, Copyright ©2013 SAS Institute Inc., Cary, NC, USA). All plots were generated in R Studio (R version 4.0.1 ^29^;) using the ggplot2 package (version 3.3.3^30^;). Anticipatory behaviour was analyzed as the total frequency of behavioural transitions/min; each time a mouse changed from one behaviour to another during the 1-min trial (following the ethogram in Table 1), this was counted as a behavioural transition. Occurrences of ‘out of view’ were excluded from this rate, and any time spent ‘out of view’ was excluded from the trial length. There were no notable differences between trials occurring in the late morning versus early afternoon, so time of day was excluded from analysis. Results were separated into baseline trials (day 1) and experimental trials (mean results for days 8–14), and the change from baseline was calculated for each mouse by subtracting baseline from experimental frequencies. Data were normally distributed and analyzed using a linear mixed model with variance components covariance structure, selected based on lowest AIC values. Treatment, strain, and treatment x strain were included as fixed effects, and cage was specified as a random effect. The effect of treatment was assessed at the cage level.

The effect of day on latency to enter the playpen was analysed using a mixed effects model with autoregressive covariance structure. The effect of strain, the linear effect of day, the quadratic effect of day, and the strain x day interaction were included as fixed effects, with cage again specified as a random effect. There was a significant interaction of strain x day (F_2,101_ = 4.81, P = 0.01), so we then re-ran the model separately by strain, treating mouse as the experimental unit.

The overall activity categories of “ambulatory” and “non-ambulatory” were used for grouping of scan sampling observations, following Draper^27^. Results for scan sampling during playpen sessions and focal sampling of agonistic interactions are presented descriptively.

## Results

### Latency to enter playpens

**Figure 2:**
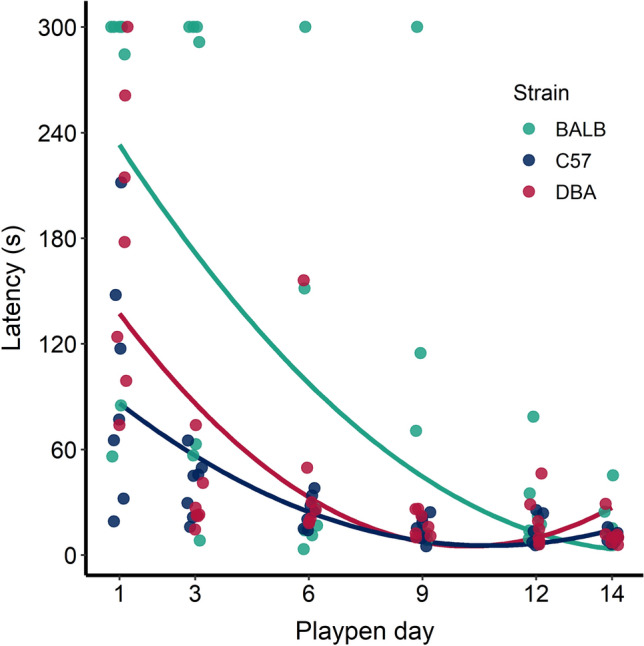
Latency (s) for each mouse to voluntarily enter the playpen. Mice are shown as individual points (horizontally jittered to avoid fully overlapping points) while genetic strains are shown in different colours. Lines depict model output; note that the quadratic term was included for all three lines for consistency in visualization, but for the BALB mice the quadratic term was not significant. The maximum possible latency was 300 s. Seven mice were tested of each strain.

Playpen mice entered the playpens more quickly with repeated testing; latency on day 1 averaged (mean ± SE) 169 ± 22.4 s, versus 13 ± 2.0 s on day 14 (Fig. 2). There was a significant strain × day interaction (F_2,101_ = 4.81, p = 0.01), reflecting that this decline in latency was more pronounced for some strains than others. The most prominent strain differences were on day 1 when the playpen was novel to mice. The BALB mice showed a high latency to enter the playpen initially (intercept = 267 ± 43.2 s); this declined with time (linear slope of − 34 ± 13.2 s/d; t_33_ = − 2.6, p = 0.01), with no statistical evidence for a quadratic effect of time. For DBA mice the latency intercept was lower (167 ± 19.9 s), with a linear slope of − 32 ± 6.6 s/d (t_33_ = − 4.79, p < 0.0001), and quadratic slope of 1.5 ± 0.4 s/d^2^ (t_33_ = 3.59, p = 0.001). The initial latency of C57 mice was lower still (intercept 103 ± 13.7 s), and again declined with day (linear slope − 18 ± 4.5 s/d; t_33_ = − 4.1, p = 0.0003), especially so during the first few days of exposure (quadratic slope 0.9 ± 0.3 s/d^2^; t_33_ = 8.67, p < 0.01). BALB mice were the only strain showing the maximum latency of 300 s after day 1, but this ended by day 12. By day 14, strain differences in latency were less pronounced (mean ± SE: BALB 17.7 ± 5.1; C57 10.1 ± 1.2; DBA 12.2 ± 2.9); all mice took less than 45 s on the last day, with the fastest mice reaching the playpen in 6 s.

### Anticipatory behaviour

**Figure 3:**
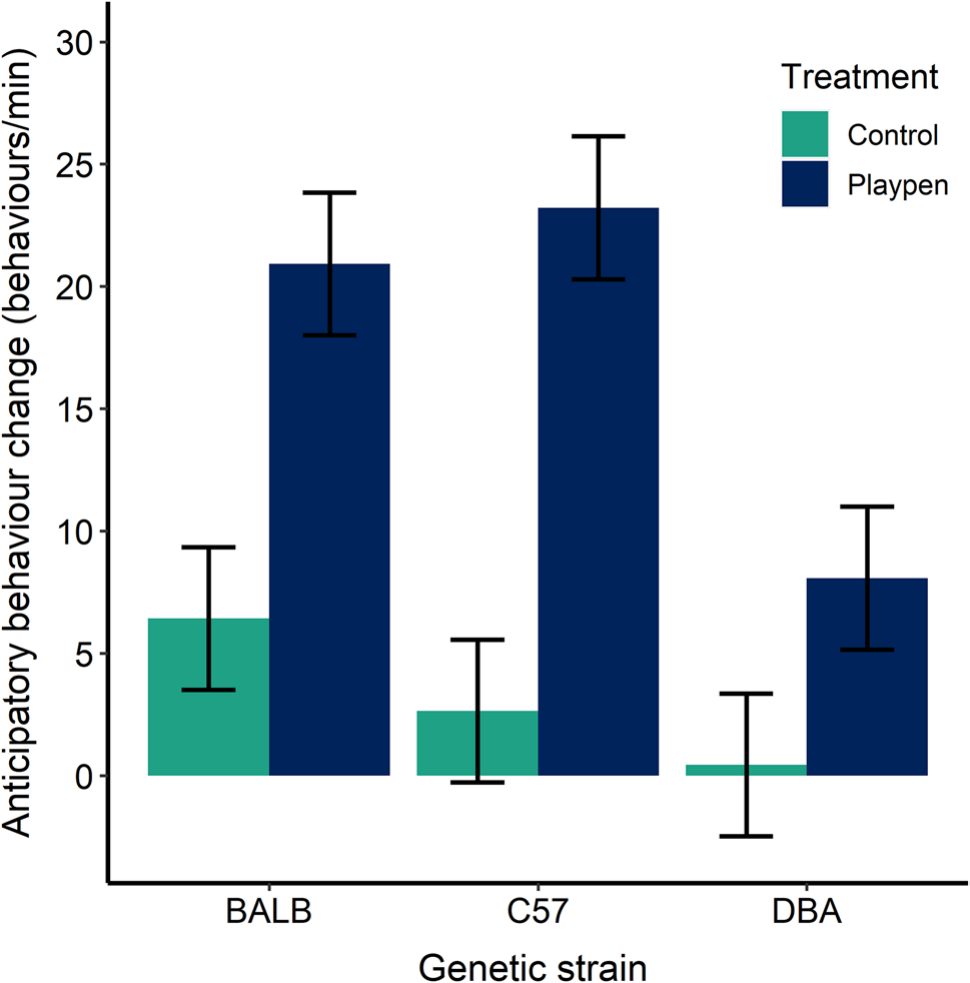
Change in anticipatory behaviour (mean behavioural frequency/min) from baseline (day 1) to experimental days (average of day 8–14). The plot shows LS means and standard error generated by the mixed model; n = 42 mice (14 mice per strain) and 7 cages per treatment.

Treatment had a significant effect on anticipatory behaviour (F_1,12_ = 35.61, p < 0.0001), with only playpen mice showing evidence of increased anticipation. When relative change was assessed (change from baseline divided by baseline values), in the playpen treatment this equated to a mean increase of 575% for BALB mice, 113% increase for C57 mice, and 81% increase for DBA mice. In the control condition, BALB mice showed a 105% increase, while C57 mice showed a 14% increase, and DBA mice showed a 4% increase. The larger rate of change for BALB mice may be due to their notably low anticipatory behaviour during baseline trials, making any increase appear comparatively large. There was also an effect of strain (F_2,24_ = 6.42, p < 0.01), driven by lower anticipatory behaviour in DBA mice relative to the other strains (Fig. 3). There was some evidence of a treatment x strain interaction (F_2,24_ = 2.46, p = 0.11), again driven by lower levels of anticipatory behaviour in the DBA mice. Anticipatory behaviour post-baseline was largely characterized by locomotion (mean ± SE = 12 ± 0.8 behaviours/min) and rearing (9 ± 0.6 behaviours/min).

### Scan sample observations

**Figure 4:**
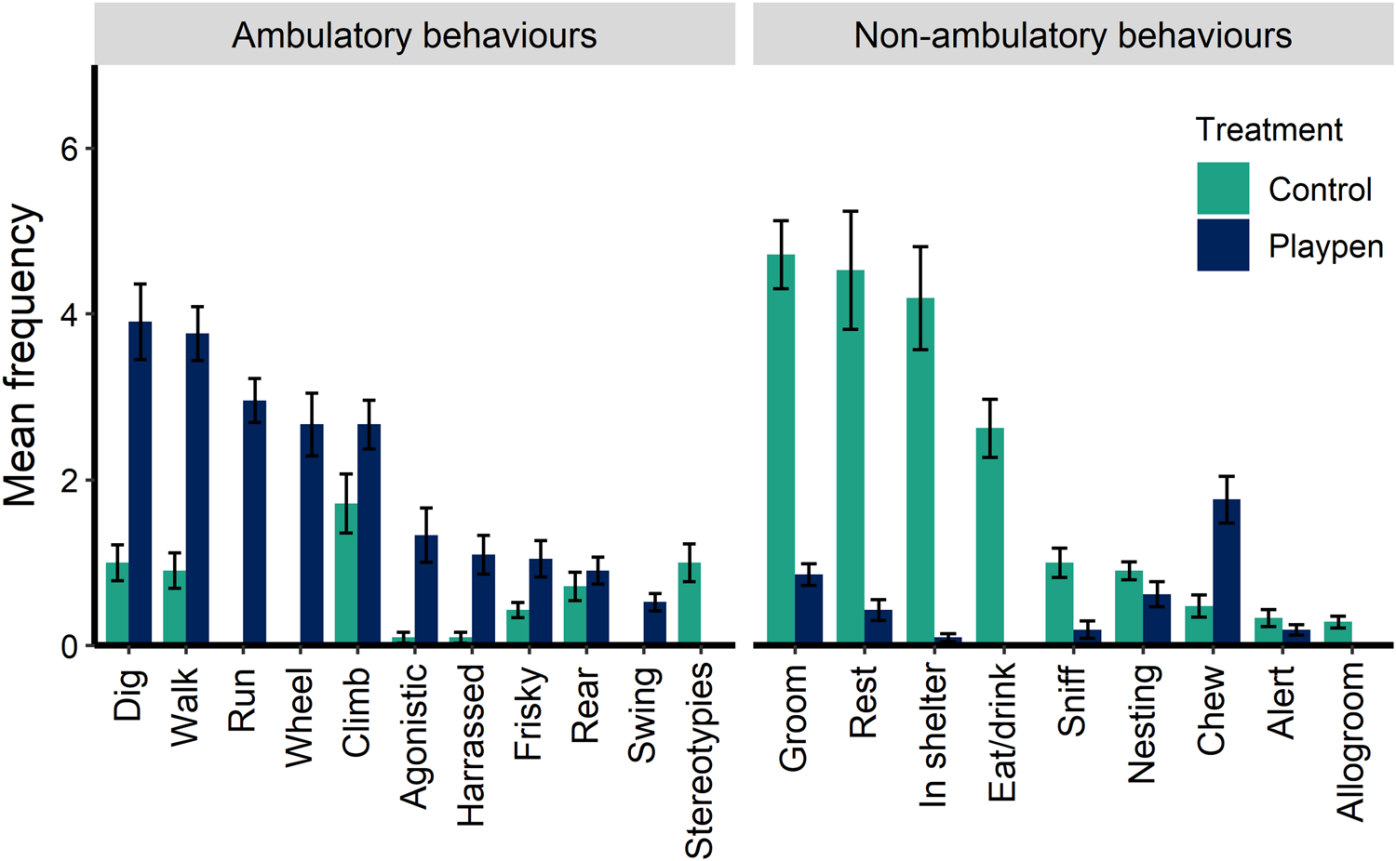
Mean (± SE) frequency of each behaviour observed during scan sampling. Behaviours are divided into the overall categories of ambulatory and non-ambulatory activity. Mice were observed 5 times per day for 5 d for a total of 25 scans per mouse; n = 42 mice, with 7 cages per treatment.

Playpen mice used both sides of the playpen about equally, with (mean frequency ± SE) 11.5 ± 1.0 vs. 13.5 ± 1.0 observations in the burrowing vs. structural sides, respectively. Control mice were observed most often in the nest (13.2 ± 1.6 observations), followed by in the open area of the cage (10.7 ± 1.5 observations), with the hut being the least utilized location (2.9 ± 0.7 observations). Playpen mice were most often observed digging, walking, running, using the wheel, and climbing, while control mice were most often seen grooming, resting, or hiding in a shelter (Fig. 4). Of the 25 observations per mouse, the mean frequency (± SE) of ambulatory behaviour was 21.5 ± 0.7 observations for playpen mice. The frequency of non-ambulatory behaviour was comparatively low at 3.5 ± 0.7 observations for playpen mice. In contrast, control mice were most frequently engaged in non-ambulatory behaviours (18.1 ± 1.1 observations). Behavioural observations are shown by strain in Supplementary Table S3.

### Agonistic interactions in the playpen

**Figure 5:**
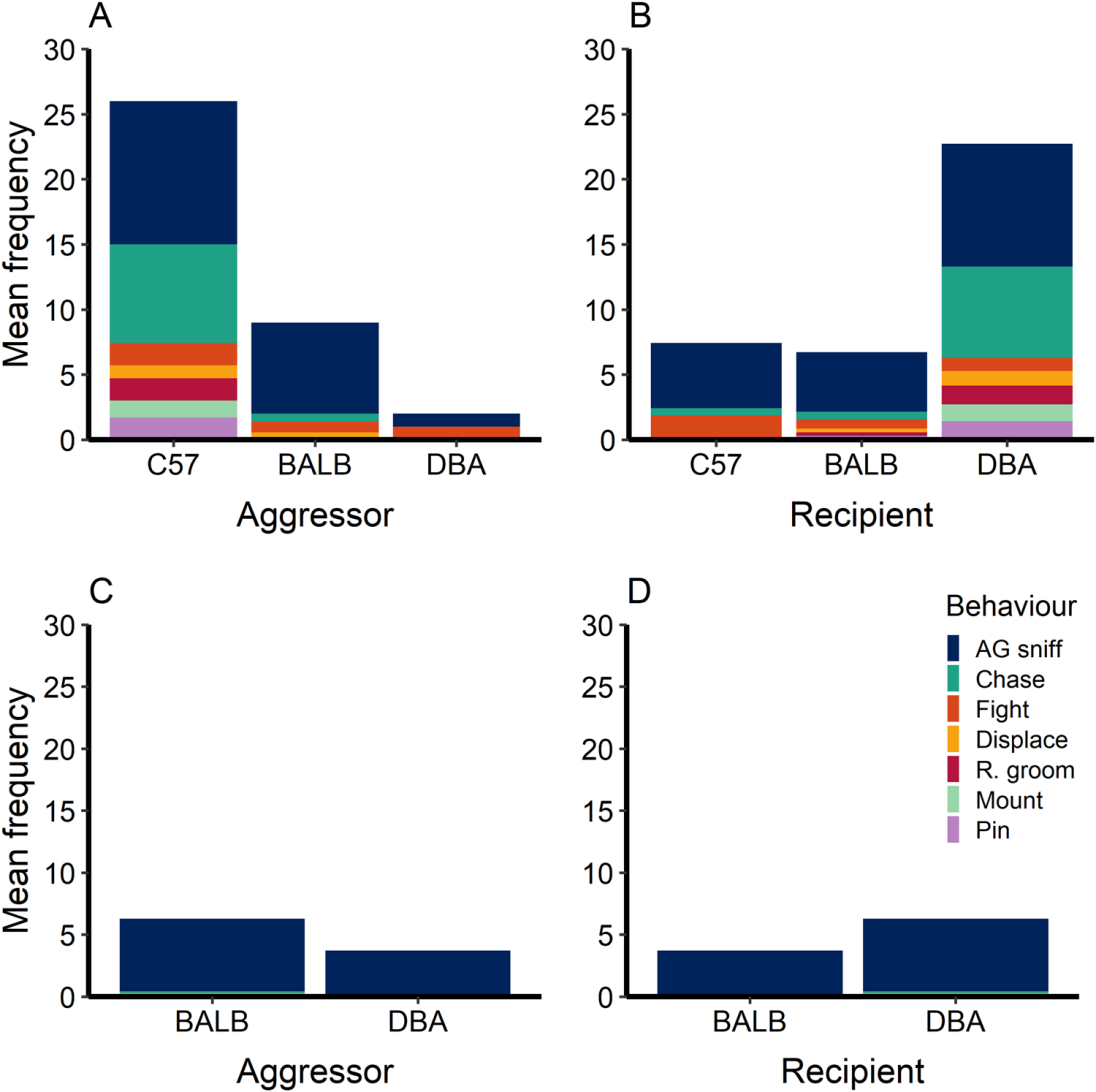
Mean frequency of agonistic behaviours observed in the playpens, shown separately by strain. Panel (**A**) shows behaviours according to aggressor strain while C57 mice were present. Panel (**B**) shows behaviours according to recipient strain with C57 mice present. Panel (**C**) shows aggressors with C57 mice absent, and Panel (**D**) shows recipients with C57 mice absent. C57 mice were present for two trials and absent for two trials. “AG sniff” stands for anogenital sniffing, and “R. groom” stands for rough grooming. Note that when mice were fighting, the mice involved were labelled as both the aggressor and the recipient. n = 21 mice, with 7 mice of each strain.

Anogenital sniffing was the most frequent agonistic interaction in the playpen. C57 mice showed the highest frequency of agonistic behaviour (mean ± SE = 26 ± 3.0), and were the only strain observed mounting, rough grooming, or pinning other mice (Fig. 5). When C57 mice were absent, BALB and DBA mice engaged in similar levels of anogenital sniffing, but did not display any fighting, displacing, rough grooming, mounting, or grooming. Sometimes, these mice showed no agonism at all; no agonistic behaviours were observed during six focal observational periods in which the C57s were absent. DBA mice showed the lowest amount of agonism (3.7 ± 0.2 with C57 mice absent; 2 ± 0.2 with C57s present) while receiving roughly three times more agonism than the other mice during observations with C57 mice present (22.7 ± 2.5, compared to 6.7 ± 1.3 for BALBs and 7.4 ± 0.9 for C57s).

## Discussion

### Latency to enter playpens

Latency to approach can be used as an indicator of how rewarding something is to an animal. Approach latency has been used with rats to infer the rewarding aspects of tickling (i.e. human-animal interaction resembling play behaviour); rats ran about four times faster to receive tickling than to receive light touch, and socially isolated rats approached the experimenter’s hand about three times faster than rats housed with social companions^19^. Differences in latency to enter an arena containing food rewards have been used to draw conclusions about motivation and reward sensitivity in mice. Spangenberg and Wichman^17^ found differences in approach latency between mice receiving a tasty reward compared to mice receiving a neutral reward. After switching from a neutral reward to a tasty reward, mice approached the reward faster than they did during baseline trials, while mice receiving neutral rewards throughout the study did not increase their speed despite having the same amount of exposure to the testing. Sherwin and Nicol^31^ found only small differences in the motivation of mice to access different sizes of additional space, indicating that mice are generally motivated to explore regardless of the size of the space offered. However, Sherwin^32^ found that mice showed higher motivation to access additional space (containing only bedding) on day 1 compared with day 2 of testing, suggesting a slight decrease in motivation once the space was less novel. In the present study, we found that mice—who had been habituated to the tunnel before the experiment—were slower to enter the playpen on the first day (likely due to neophobia), and then entered more quickly over time, with some mice running through the transfer tunnel in as little as 6 s by day 14.

There were strain differences in initial latency to enter the playpen; BALB mice were most hesitant, while C57 mice were the fastest to enter. Neophobic mice spend less time interacting with enrichment and dwelling in an unfamiliar enriched cage^20^, and therefore may also be less likely to experience its rewarding properties. Only the BALB mice reached the 300 s latency limit after day 1, while C57 mice were more willing to enter the playpens on day 1, and all entered in approximately 60 s or less by day 3. BALB mice show high baseline anxiety levels^33^ and prefer familiar environments, whereas C57 mice do not discriminate between novel and familiar environments^34^. Unfortunately, we did not record latency to enter a separate control condition, so we cannot draw conclusions regarding how motivated mice were to enter playpens compared to another environment.

### Anticipatory behaviour

Anticipation is commonly assumed to represent a state of “wanting”^35^. Elevated levels of anticipatory behaviour have been linked with activation of reward centres (i.e. opioid systems) in the brain, as demonstrated in studies where a lose dose of naloxone (an opioid blocker) attenuated the increase in behavioural transitions seen during anticipation (summarized in^36^). Previous studies with rats also support that anticipatory behaviour is indicative of rewarding properties. For example, van der Harst^16^ found that the rate of behavioural transitions shown by rats prior to accessing an enriched cage was comparable to the rate of anticipatory behaviour shown for sexual contact. They also found that rats anticipating a forced swim session did not mount an anticipatory response, while rats anticipating transfer to an enriched cage showed a significant increase in anticipatory behaviour. Another study has shown that socially stressed rats lacked an anticipatory response to a sucrose reward, but when given regular access to enriched cages, these rats began to show anticipatory behaviour for access to the enriched cage. The authors suggested that environmental enrichment may be a superior reward to sucrose, or that repeated exposure to enrichment had a therapeutic effect on depressive states^37^.

Our mice displayed increased anticipatory behaviour following a cue associated with access to a playpen, indicating that playpen access was rewarding. To our knowledge, anticipatory behaviour as a measure of reward sensitivity and emotional states has only been used in one other study with mice^17^; this study found increased anticipatory behaviour when mice were given a cue associated with a food reward. Anticipatory behaviour testing is more commonly used with rats and other species, and is most commonly quantified and reported in terms of ‘frequency of behavioural transitions’ rather than differences in specific behaviours (see^35^ for a review). Given that this measure is non-specific and has not been used extensively with mice, we encourage further validation of anticipatory behaviour as a measure of reward using cues indicating known positive and negative stimuli, as well as further analysis of the specific behaviours shown. DBA mice tended to show less anticipatory behaviour than the other two strains. It is possible that genetic disposition played a role in anticipatory behaviour, but this result also raises the question of whether DBA behaviour was influenced by the aggression received from C57 mice while in the playpen. Mixed-strain housing provided advantages, such as making individual mice identifiable, but this housing method may have influenced the behaviour of DBA mice. The results of an earlier study found that while mice were generally motivated to access enrichment, there was individual variability in this motivation^18^, suggesting that the rewarding properties of enrichment can vary among mice.

### Scan sampled behaviour

Mice in the playpens were most frequently engaged in ambulatory behaviour, with digging, locomotion, wheel use, and climbing being the most often observed behaviours. The swing was one of the least used objects, suggesting that it could be removed or replaced with something more engaging. We conclude that mice in the playpens were active, showing behaviors that are impossible or difficult to perform in the conventional cage, such as digging using both front and back legs, wheel use, and running. This has implications for welfare and animal research outcomes, given that voluntary exercise has health impacts such as increased volume in regions of the brain related to learning and spatial memory^38^, weight control^39^, reduced tumor growth^40^ and reduced anxiety^41,42^. Therefore, a higher rate of ambulatory behaviours may be associated with better welfare. These observations also demonstrate that mice were engaging with the provided objects and that playpens provided opportunities to express natural behaviour, which is consistent with literature reporting that enrichment increases activity and exploratory behaviours^43^. We did not assess if behaviour in the home cage differed between treatments during the dark period, but other studies have shown that mice in conventional cages generally spend more time resting or engaged in inactive-but-awake behaviour^11,43^.

Mice in control cages engaged in non-ambulatory behaviours most frequently, with grooming, resting and hiding within the shelter being the most frequent. Bailoo et al.^15^ reported that mice housed in barren cages spent much of their time performing maintenance behaviours such as grooming. Maintenance behaviours decrease when mice are housed in cages with basic enrichment components (bedding, nesting material, a tunnel, and a shelter), and decrease even further when mice are housed in cages containing more complex enrichment components (e.g. larger cages with platforms, ladders, hammocks, nesting material, shelters, and tunnels), indicating that grooming may be a type of displacement behaviour expressed when other natural behaviours are unavailable to mice^15^. Mice in the playpens were rarely observed grooming.

Previous studies have reported that laboratory mice will quickly begin to dig if provided with an appropriate substrate^3,4^. Indeed, the high level of digging seen in the current study suggests that mice are motivated to burrow. Mice in the control cages were sometimes observed digging in their bedding, but in a conventional cage digging typically involved only the mouse’s front legs rather than the rear legs, and the shallow cage bedding could not be manipulated to form tunnels. Mice are willing to work for access to burrowing substrate and perform this behaviour consistently, regardless of whether they have access to an intact burrow^4^. Access to burrowing substrate is not often considered in mouse enrichment experiments; our results suggest that this may be an important activity for mice. Mice in this study burrowed in both substrates (soil and coconut fibre), suggesting that both options are suitable for future use. Coconut fibre may be preferred in some facilities as it is generally easier to sterilize and manage.

The burrowing substrate and structural enrichment sides of the playpen were used about equally, which may indicate that access to a variety of behavioural opportunities is valuable to mice. However, we did note some evidence of individual or strain preferences. For example, the DBA mice were most frequently observed digging in the playpens, while C57 mice were most frequently observed walking or running, and BALB mice were most frequently observed walking or using the running wheel (see Supplementary Table S3 for summary of playpen behaviours by strain).

### Agonistic behaviour in the playpen

Agonistic interactions were relatively common in the playpen and were largely initiated by C57 mice; there were observational periods where no agonism occurred, when C57 mice were absent. Female mice typically show decreasing agonism when enrichment is incorporated into cages^11^; however, enrichment in our study was provided in a larger temporary environment outside the home cage, and we observed the opposite trend. The majority of agonism was shown via anogenital sniffing; other more overtly dominant or aggressive behaviours such as mounting, pinning, and fighting were only performed by C57 mice. There were no visible injuries resulting from these interactions. Because we did not sample the playpen mice in their home cages, we cannot conclude whether this level of agonistic interaction was typical of home cage dynamics or whether it was related to the playpens per se.

It is not clear to what extent aggression and territorial behaviour are appetitive for mice, although the lower anticipatory behaviour of DBA mice prior to playpen access suggests that being the recipient of this behaviour may have been aversive. Aggression is an important component of the behavioural repertoire of rodents. Laboratory mice exhibit aggression for a variety of reasons, such as frustration, stress, insufficient enrichment, or impaired social skills^44^. There is also an appetitive component of aggression (for the aggressor); Golden et al. demonstrated that aggressive mice developed a conditioned place preference for a location where they were able to attack an intruder mouse^45^. In another study using female Syrian hamsters, aggressive encounters resulted in behavioural and neurological changes associated with rewarding effects^46^. In the current study, the playpen allowed for expression of more agonistic behaviours, notably chasing initiated by C57 mice, which cannot occur to the same extent in shoebox cages. The nature of territorial or aggressive behaviour may be altered in conventional cages because mice cannot fully express the behaviour and achieve the desired result (i.e. removal of the subordinate mouse from the territory)^44,47^. The chasing we saw from C57 mice in the playpens may be a result of these mice being able to exhibit a motivated natural behaviour that is thwarted in conventional cages, albeit at the expense of the more docile DBA mice. C57 mice have been classified as highly social^48,49^, but in reality this may be due to a motivation to exert dominance over other mice.

### Limitations and future directions

The current study provided playpen access during the light period (rather than during the dark period when mice are naturally more active), as we believe that this is likely how playpens would be used within research facilities. Providing playpen access when mice would normally be sleeping is a limitation of the study; we suggest that motivation for playpen access would be greater if this was offered in the dark period. Mice in the playpens still chose to engage in active behaviours; if left in the playpens for longer durations, we expect that mice would have formed nests or burrows to rest in. We also would have likely observed more ambulatory behaviour in control mice during the dark period.

The aim of the present study was not to identify the value of specific enrichment components; rather we were interested in whether temporary access to a larger and more complex environment was perceived positively by conventionally housed mice. Thus, we cannot parse out the specific effects of space, physical environmental components, smells, treats, or voluntary vs. non-voluntary access. Future research could disentangle specific aspects of the playpen experience, such as mouse preferences and motivation for specific components. However, while studying a singular enrichment item can provide clarity on animal preferences and the effects of specific cage additions (e.g. ^50,51^), providing single items has been shown to be less effective at improving welfare compared to the provision of more extensive enrichment. Other work has concluded that more complex conditions (including climbing structures, tunnels, shelters, wheels, nesting material, elevated platforms, hammocks, and bridges) were most impactful in terms of animal welfare outcomes, even when compared to mice housed with basic enrichment components (bedding, nesting material, a tunnel, and a shelter ^15^;). The ability to engage with a variety of enrichment components may be more valuable to animals; for example, rats provided with five different enrichment items within their cage displayed more signs of improved welfare than rats provided with only one item^52^, or five copies of any one item^53^.

Generally mice may perceive handling as aversive^54^. In the present study, aversion was unlikely to be caused by the transfer method used; mice were given the opportunity to travel between the playpen and home cage on their own, and any handling (non-voluntary playpen entry or exit) was done using overturned huts or tunnels. If mice are handled during transfer (rather than using our tunnel technique), then we suggest that using low-stress handling methods is important. The use of physical handling to move mice in and out of playpens may negatively affect their overall experience. We also suggest that future work should assess playpen use with male mice, given that playpens could be aversive if aggression in males is more common.

We only assessed mouse behaviour during playpen access, so conclusions can only be made according to this time period. Stereotypies were only seen in control mice and never within the playpens, possibly due to differences in the structural environments provided (e.g. bar-biting was not possible in the playpens). It is expected that mice in the control cages would be more active during the dark period; however, it is also expected that most ambulatory behaviour in conventional cages would be expressed in the form of stereotypies. Future work should assess whether playpen access impacts the development of stereotypies in mice.

Enrichment is generally provided to improve mouse welfare^2^, and this was our goal in providing playpen access. However, it is possible that playpen access might also result in worse overall welfare. For example, a study by Latham and Mason^55^ presented evidence that losing access to enrichment led to frustration in mice, shown through exacerbated stereotypic behaviours. These authors also found increased corticosterone in male mice transitioned from enriched to conventional housing. It is not clear if temporary access as provided in the current study would have the same effects, but these results suggest that mouse welfare is likely to be compromised if regular access is discontinued. Future work should investigate the possibility that conventional home cages may become more negative to temporarily enriched mice, and whether playpen access can be managed to avoid this outcome. Access three times per week for 30 min at a time was enough to evoke a clear change in anticipatory behaviour and latency entering the playpens; it is not clear what frequency or duration is optimal for other welfare outcomes. To further understand the potential impact of social dynamics between different strains on our outcomes, we suggest future work using single-strain comparison cages, as well as a quantitative measure of motivation such as maximum price paid (MPP) for access (e.g.^4,18^) to assess if mice receiving aggression have a lower MPP than aggressor mice.

There are pros and cons of this approach to environmental enrichment. We suggest that playpens could be a feasible solution for facilities unable to increase the size or complexity of home cages. Playpens may be a particularly feasible option for rodent colonies that are kept for teaching or sentinel purposes. This method also allows for the provision of enrichment opportunities that would not fit within a conventional cage (such as a deep burrowing substrate). In our case, we were not concerned about biosecurity risks between cages within our colony, but for some this may be a concern. The playpens remained reasonably unsoiled for the duration of the study and required little maintenance. To reduce workload on animal care staff, researchers can also place their animals in playpens for short durations while in the facility (as was done in^56^, for example). If human exposure to allergens is a concern, individually ventilated rat cages could be used; the cages used in the present study can be connected to a rack to provide ventilation.

## Conclusions

We found that (1) mice entered playpens more quickly over time, (2) mice showed increased anticipatory behaviour before accessing the playpen, and (3) mice in the playpen showed more ambulatory behaviours compared to control mice that mainly performed non-ambulatory behaviours such as resting or grooming. Mice in the playpen displayed a range of agonistic behaviours, most commonly anogenital sniffing or chasing; overt aggression (i.e. fighting, pinning) was observed more rarely and was typically initiated by C57 mice. Voluntary playpen access is rewarding to female mice and allows for the expression of natural behaviours; playpens may be an effective method of providing access to increased environmental complexity in laboratories.

## Supplementary Information

Supplementary Information.

## Data Availability

Datasets are available from the Figshare database (URL: https://figshare.com/projects/Mouse_playpen_data_Ratuski_et_al_2021_/99728).
