# Supplementary material for: Using approach latency and anticipatory behaviour to assess whether voluntary playpen access is rewarding to laboratory mice
Source: Sci Rep. 2021 Sep 21;11:18683. doi: 10.1038/s41598-021-98356-3 (PMC8455539; doi:10.1038/s41598-021-98356-3)
Supplement: Supplementary file 1 — Supplementary Information. [file 41598_2021_98356_MOESM1_ESM.pdf]

Using approach latency and anticipatory behaviour to assess whether voluntary playpen access is rewarding to laboratory mice

Anna S. Ratuski[1]*, I. Joanna Makowska[1], Kaitlyn R. Dvorack[1], Daniel M. Weary[1]
[1]Animal Welfare Program, Faculty of Land and Food Systems, 2357 Main Mall, University of British Columbia, Vancouver, BC, V6T 1Z6, Canada

Supplementary Information

Supplementary methods: Pilot study description.

A pilot study was conducted to determine if mice were willing to use playpens during the light period, which enrichment objects they found engaging, and to refine the methods used for data collection in the main study (e.g., anticipatory behaviour testing and behavioural scan sampling). Mice used in the pilot study were diverse: they were acquired from another researcher as surplus stock, and thus varied in sex (n= 28 females; n=27 males), strain (DHHC5, Del7AI, and BoyJ), and age (ranging 2-8 months). These mice were housed in standard cages (see description in the main experiment) in single-sex groups ranging from 3-5 mice. Cages were randomly allocated to the playpen (n=28) or control (n=27) treatment. Latency to enter the playpen was not measured, and soil was the only burrowing substrate provided.

The pilot study allowed us to determine that some items in the playpen were unused (e.g. wooden chewing blocks) and thus could be replaced with potentially more engaging items. We also noted that mice preferred upright vs. disc-like running wheels. We were also able to identify the best filming angles, adjust methods for transferring mice to and from playpens, and determine a feasible live scan sampling schedule. We also found that male and female mice behaved differently in the playpens; males appeared to display more complex social dynamics, therefore we decided to focus on each sex in separate experiments, starting with females.

Supplementary Table S1. Habituation to the tunnel used for playpen access. On day 1 mice were given 10 min of exposure to the tunnel in their home cage. On day 2 and 3 mice were given 5 min of exposure. Fully crossing the tunnel refers to a mouse entering one side and exiting the other side at least once during the session.

Cage	Strain	***Habituation Day***					
		1		***2***		***3***	
		Entered tunnel	***Fully crossed tunnel***	***Entered tunnel***	***Fully crossed tunnel***	***Entered tunnel***	***Fully crossed tunnel***
2	BALB	No	No	No	No	No	No
	C57	Yes	Yes	Yes	Yes	Yes	Yes
	DBA	Yes	Yes	Yes	No	Yes	Yes
4	BALB	Yes	No	Yes	Yes	Yes	Yes
	C57	No	No	Yes	Yes	Yes	Yes
	DBA	Yes	Yes	Yes	Yes	Yes	Yes
6	BALB	Yes	No	Yes	Yes	Yes	Yes
	C57	No	No	Yes	Yes	Yes	Yes
	DBA	Yes	Yes	Yes	Yes	Yes	Yes
8	BALB	No	No	Yes	No	Yes	No
	C57	Yes	Yes	Yes	Yes	Yes	Yes
	DBA	Yes	Yes	No	No	Yes	Yes
10	BALB	No	No	No	No	No	No
	C57	Yes	Yes	Yes	Yes	Yes	Yes
	DBA	No	No	No	No	Yes	Yes
12	BALB	Yes	Yes	Yes	Yes	Yes	Yes
	C57	No	No	No	No	Yes	Yes
	DBA	No	No	Yes	No	Yes	Yes
14	BALB	Yes	No	Yes	Yes	Yes	Yes
	C57	No	No	Yes	Yes	Yes	Yes

	DBA	No	No	Yes	No	Yes	Yes

Supplementary Table S2. Training schedule for anticipatory behaviour trials. Outcome refers to what happened following the waiting period; for playpen mice the outcome was playpen access while for control mice the outcome was putting their home cage back on the ventilated cage rack. The maximum waiting time was 60 s, which was reached on playpen day 8 and was repeated until day 14.

Playpen day	Time between cue and outcome (seconds)
**1**	60
**2**	0
**3**	10
**4**	20
**5**	30
**6**	40
**7**	50
**8-14**	60

Supplementary Table S3. Mean frequency ± SE of each behaviour performed by mice while in the playpens, shown separately by strain. Mice (n=21) were observed a total of 25 times.

Behavior	BALB	C57	DBA
**Agonism**	0.3±0.1	3.4±0.4	0.3±0.1
**Alert**	0.4±0.1	0±0	0.1±0.1
**Allogroom**	0±0	0±0	0±0

**Climb**	2.6±0.3	3.7±0.4	1.7±0.2
**Chew**	1.9±0.3	0.9±0.1	2.6±0.3
**Dig**	1.6±0.1	4±0.5	6.1±0.4
**Eat/drink**	0±0	0±0	0±0
**Fight**	1±0.1	0.7±0.2	1.4±0.3
**Groom**	1±0.1	0.4±0.1	1.1±0.1
**Harassed**	0.9±0.2	0.3±0.1	2.1±0.3
**Run**	2.1±0.2	4.1±0.3	2.6±0.2
**Walk**	4.4±0.4	4.6±0.2	2.3±0.2
**Nesting material**	0.9±0.1	0.1±0.1	0.9±0.2
**Rear**	2±0.2	0.3±0.1	0.4±0.1
**Rest**	0.9±0.2	0.1±0.1	0.3±0.1
**In shelter**	0.1±0.1	0±0	0.1±0.1
**Sniff**	0.6±0.2	0±0	0±0
**Stereotypies**	0±0	0±0	0±0
**Swing use**	1±0.1	0.3±0.1	0.3±0.1
**Wheel**	3.4±0.4	2±0.4	2.6±0.4

Supplementary Discussion: Use of transfer tunnels for playpen entry and exit.

We found the transfer tunnels to be a practical method that allowed for increased animal agency, reducing the amount of handling required. On days 20 and 21 of playpen access, we recorded latency to exit playpens through the tunnels. Once habituated to the tunnels and the playpens, mice took on average 15 ± 3.8 s to return to their home cages after playpen access; the fastest

mouse required only 4 s and the slowest took 84 s. This ease of transfer was likely aided by providing mice with a food reward in their home cage following playpen access.
